# Exact direct-space asymmetric units for the 230 crystallographic space groups

**DOI:** 10.1107/S0108767311007008

**Published:** 2011-03-31

**Authors:** Ralf W. Grosse-Kunstleve, Buddy Wong, Marat Mustyakimov, Paul D. Adams

**Affiliations:** a Physical Biosciences Division, Lawrence Berkeley National Laboratory, 1 Cyclotron Road, BLDG 64R0121, Berkeley, California, 94720-8118, USA; b UC Leads Summer Research Program, University of California, Berkeley, California, 94720, USA; c Bioscience Division, Los Alamos National Laboratory, B-8, MS M888, Los Alamos, New Mexico, 87545, USA

**Keywords:** asymmetric unit, direct space, space groups

## Abstract

It is well known that the direct-space asymmetric unit definitions found in the *International Tables for Crystallography*, Volume A, are inexact at the borders. Face- and edge-specific sub-conditions have to be added to remove parts redundant under symmetry. This paper introduces a concise geometric notation for asymmetric unit conditions. The notation is the foundation for a reference table of exact direct-space asymmetric unit definitions for the 230 crystallographic space-group types. The change-of-basis transformation law for the conditions is derived, which allows the information from the reference table to be used for any space-group setting. We also show how the vertices of an asymmetric unit can easily be computed from the information in the reference table.

## Abstract 2

A reference table of exact direct-space asymmetric units for the 230 crystallographic space groups is presented, based on a new geometric notation for asymmetric unit conditions.

## 1. Introduction   

In the presence of symmetry, the concept of an *asymmetric unit* (also known as *fundamental region* in mathematics) is important for many practical applications, for example to avoid time-consuming redundant calculations or to suppress redundant output. *International Tables for Crystallography*, Volume A (ITA) (Hahn, 2005 ▸) defines the direct-space asymmetric unit of a crystallographic space group (DAU) as ‘the smallest part of space from which, by application of all symmetry operations of the space group, the whole of space is filled exactly’ (ITA §2.2.8). This paper focuses on definitions of exact DAUs. These are refinements of the ITA conditions, which are sets of inequalities for each space group that must be true simultaneously for a point with fractional coordinates 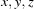 to be inside the DAU, for example 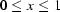; 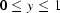; 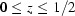 for space group 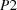. The ITA conditions define the DAU shapes but are inexact for the borders. For example, the ITA 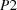 conditions are true for all eight vertices of the DAU parallelepiped, but according to the definition in ITA §2.2.8 only two of these points can be in the exact asymmetric unit. To make a DAU exact, sub-conditions have to be added to the shape conditions, specific to faces and edges. Koch & Fischer (1974 ▸) published exact DAU definitions for the cubic space groups. In Grosse-Kunstleve *et al.* (2003 ▸) we presented an overview of an online gallery of exact DAUs for all 230 crystallographic space groups. Chapter 1.5 of Shmueli (2008 ▸) and the KVEC server at http://www.cryst.ehu.es also offer exact DAU definitions, but the DAU shapes are partially incompatible with those of ITA. In this paper we introduce a concise geometric notation which is the foundation for a reference table of exact DAUs, using the same definitions as in our previous work, which are fully compatible with those of ITA.

## 2. Geometric cut notation and expressions   

This section defines a concise geometric notation that has greatly accelerated the progress of this work. As will become apparent below, the notation enables a systematic, intuitive labelling of planes that define an exact DAU.

**Figure 1:**
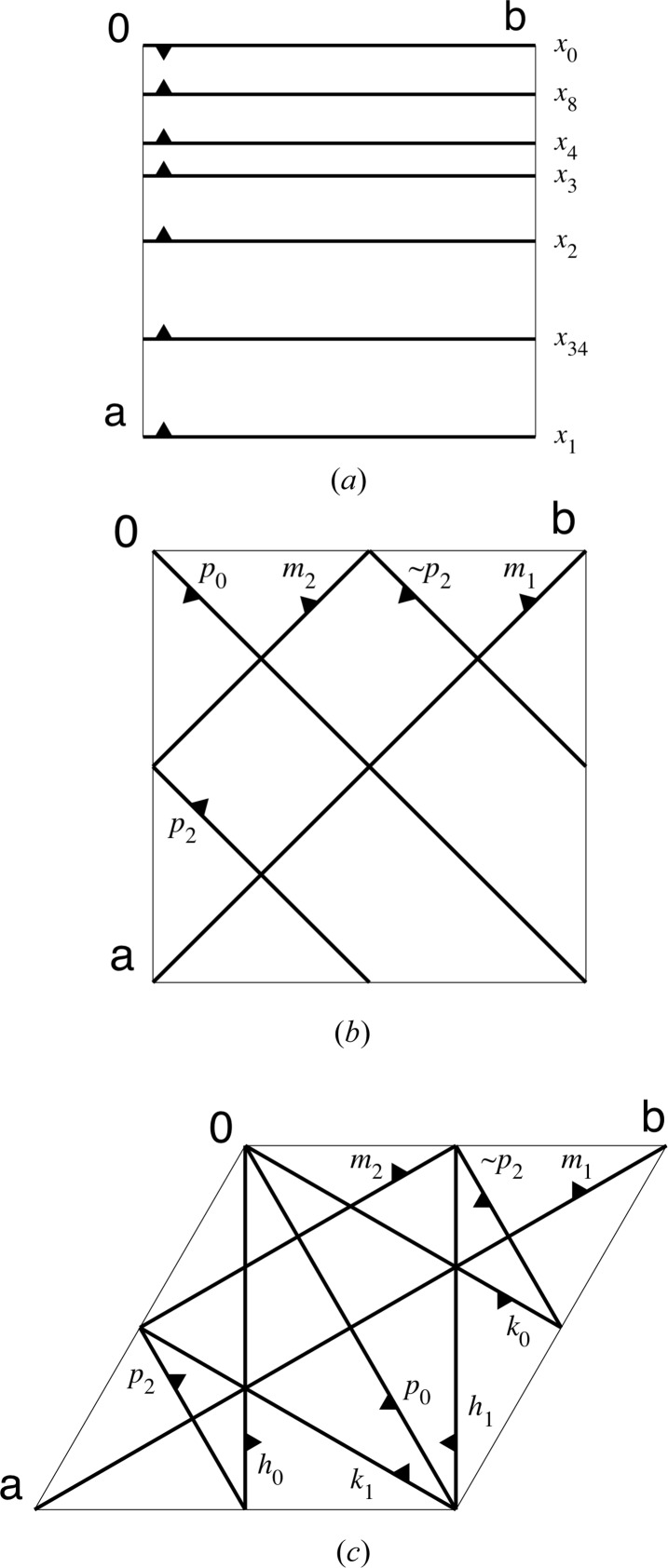
Illustrations of selected cut planes with geometric cut symbols *x*_*d*_, *p*_*d*_, *m*_*d*_, *h*_*d*_ and *k*_*d*_. The (*x*, *y*, 0) plane of a unit cell is outlined with thin lines. The unit-cell origin is labelled with **0**, the basis vectors with **a** and **b**. The traces of cut planes are indicated with thick lines; small attached triangles indicate the ‘inside’ direction. (*a*) Geometric interpretation of equations (7) and (8) using the *x*_*d*_ flock as an example. (*b*) Cut planes in the *p*_*d*_ and *m*_*d*_ flocks as they appear in a tetragonal system. The *p*_2_ and ~ *p*_2_ cut planes illustrate the geometric interpretation of equation (6). (*c*) Cut planes in the *p*_*d*_, *m*_*d*_, *h*_*d*_ and *k*_*d*_ flocks as they appear in a trigonal or hexagonal system.

Similar to the ITA approach, a DAU shape is defined by a list of inequalities. We work with the general form 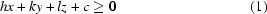
or 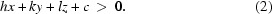


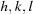
 are Miller indices that define the normal vector of a plane, 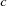 is a scalar constant which determines the distance of the plane from the origin, and 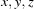 are fractional coordinates in direct space. We call both equations a *cut* since the geometric interpretation is a division of direct space into two halves. The left-hand side of the equations is exactly zero for points inside the cut plane. The inequalities are defined to be true for points 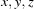 inside the DAU. Equation (1) is used if a region of the cut plane is inside the DAU and equation (2) is used if the entire plane is outside. To facilitate a concise representation of DAU definitions, we introduce a *cut notation*. The general form is 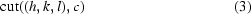
or
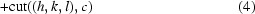
corresponding to equations (1) and (2), respectively. To obtain intuitive labels for DAU cut planes, we use the *geometric cut symbols* defined in Table 1 ▸, for example 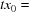 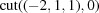. Relationships between cuts can be formalized *via cut expressions* using unary and binary operators defined as follows: 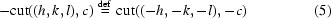


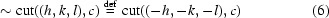


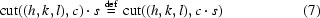


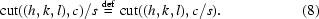
The variable 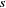 is a scalar value. Each of the operators defined in equations (5) [Disp-formula fd6] [Disp-formula fd7]–(8) has a simple geometric interpretation. The ‘−’ operator defined by equation (5) corresponds to a reversal of ‘inside’ and ‘outside’. The ‘~’ operator defined by equation (6) acts like a centre of inversion at the origin; see Figs. 1 ▸(*b*) and 1 ▸(*c*) for an example. The multiplication and division operators defined by equations (7) and (8) provide a notation for parallel shifts, as highlighted by Fig. 1 ▸(*a*).

The DAU conditions of ITA have a straightforward correspondence to our cut definition. We call the ITA conditions *shape cuts*. We employ the concept of context to avoid redundancy in the definition of sub-conditions specific to a given DAU face by appending the sub-conditions to the corresponding shape cut, surrounded by parentheses; such a cut is a *face cut*. Similarly, sub-conditions specific to a given edge are appended to a corresponding face cut, again surrounded by parentheses, and are called *edge cuts*. In some cases, the DAU choices of ITA necessitate the combination of cuts *via* logical conjunction or disjunction. Following common practice, we chose the symbol ‘&’ for conjunction and ‘|’ for disjunction. To give an example, the cut expression 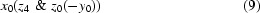
appears for space group 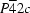 (No. 112), using the geometric cut notation of Table 1 ▸. Here 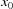 is a shape cut. The expression in the outer pair of parentheses is a face cut, composed of the logical conjunction *z*_4_ & *z*_0_. The expression in the inner pair of parentheses (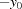) is an edge cut. As an example, a full step-by-step interpretation of equation (9) is shown in Appendix *A* [App appa].

The symbols in Table 1 ▸ include seven main flocks of parallel planes: *x*_*d*_, *y*_*d*_, *z*_*d*_, *p*_*d*_, *m*_*d*_, *h*_*d*_, *k*_*d*_. The position of a cut plane relative to the origin of the coordinate system is indicated with the index *d* = 1/*c*, with *c* as defined in equations (3) and (4), except if *c* = 0 or *c* = 3/4. Fig. 1 ▸ illustrates the geometric interpretation of the main geometric cut symbols. A large majority of the cut planes needed in the DAU definitions presented below can be labelled intuitively with these symbols. The remaining symbols in Table 1 ▸ were introduced primarily to condense the DAU definitions in Table 2 ▸ below.

## 3. Methods   

### 3.1. Change-of-basis transformation law   

In many situations it is essential to be able to transform variables from one basis system to another. Giacovazzo (1992 ▸) includes a table of transformation laws (Table 2.E.1) for commonly used variables, for example fractional coordinates, Miller indices or anisotropic displacement parameters. This list can be extended by a transformation law for DAU definitions based on equations (1) and (2). Borrowing the conventions of ITA, let (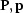) be a change-of-basis matrix with a (3 × 3) rotation part 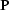 and 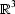 translation vector 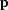, and let (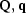) be its inverse. A column vector of fractional coordinates 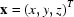 in a first 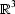 basis system is transformed to coordinates 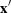 in a second basis system *via*

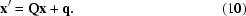
We also define the row vector 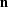 = (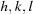) in the first basis system. The corresponding 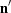 in the second basis system is given by 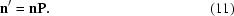
The determination of the scalar constant 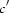 is based on the rationale that 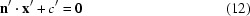
must hold for all solutions 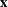 of 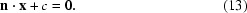
Setting 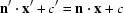, substituting equations (10) and (11), and solving for 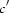 yields the second part of the transformation law for DAU cuts: 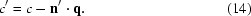



### 3.2. Determination of vertices   

Given a list of shape cuts, the DAU vertices can be computed by solving equation (13) for all unique ordered triplets of cuts. Let 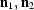 and 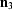 be the cut normal vectors of such a triplet. The three cut planes intersect in a point 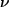 if the determinant of 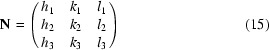
is not zero. Under this condition the point 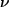 is found by solving 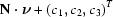 = 0: 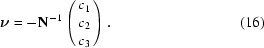


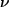
 is a vertex of the DAU if all inequalities given by the shape cuts are also simultaneously true. If more than three planes intersect in a given vertex it is obtained multiple times and duplicates are discarded. We note that the largest number of shape cuts using the ITA definitions is nine, for space group 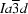 (No. 230). In this case the determinant of 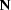 is evaluated 84 times, equation (16) is evaluated 56 times and the final number of unique vertices is nine, in accordance with ITA.

### 3.3. Validation of exact conditions   

The exact conditions shown in Table 2 ▸ are validated with a sampling procedure to establish that the DAU is neither too small nor too large. The procedure is intentionally unsophisticated to maximize robustness. It is intrinsically highly inefficient, which is compounded by the use of a dynamically typed scripting language for its implementation. Nonetheless, given current computing hardware, the entire Table 2 ▸ can be re-validated in less than 2 min.

The first part of the validation procedure samples the DAU conditions using two grids over the unit cell, given a user-defined number of sampling points *N* per unit in fractional coordinate space. To simplify this presentation, without loss of generality, we assume that *N* is identical in all three dimensions. *N* is always chosen to be even. The first *ugrid* covers the unit cell from 0 to *N* − 1, corresponding to the range [0.0, 1.0[ in fractional coordinate space. All *ugrid* points are initialized with zero. The second *rgrid* covers space more redundantly from −*N*/2 to *N*, corresponding to the range [−0.5, 1.0]. The vertex determination of §3.2 is used to assure that the DAU to be validated falls entirely into the *rgrid*. For each *rgrid* point, the inequalities defined by the DAU cuts are evaluated. A value of one is assigned if the point is inside the DAU (all inequalities are true) and zero otherwise. If the point is inside the DAU, the crystallographic unit translations, in the form of the modulus operation, are applied to the grid indices of the point to determine the symmetry-equivalent grid point in the *ugrid*, which is then also set to one. If it was set already, an error message reports that the point is redundant.

At the end of the first part of the validation procedure the *ugrid* has a value of one for all grid points inside the DAU and zero for all points outside; note that the *ugrid* has disconnected regions of grid points with value one if the DAU has points with negative coordinates. The second part of the validation procedure visits each point in the *ugrid*. The symmetry operations of the space group, taking the crystallographic unit translations and any centring translations into account, are applied to enumerate all equivalent points in the *ugrid*. If a point is flagged as inside the DAU, all equivalent points must be flagged as outside; otherwise an error message reports that the DAU has redundant points. For each point flagged as outside the DAU, one equivalent point must be flagged as inside; otherwise an error message reports that the point has no equivalent in the DAU.

If no error messages are shown, the validation procedure establishes conclusively that the DAU conditions have complete coverage and that the covered space is non-redundant under symmetry. The only critical parameter is the number *N* of sampling points per unit in fractional space. Based on an inspection of the locations of the symmetry elements, we found that *N* = 24 is sufficiently large for all space groups. However, as a final validation we also ran the procedure for all space groups with *N* = 72, which takes about 45 min on a current 48-core system.

### 3.4. Visually assisted determination of exact conditions   

**Figure 2:**
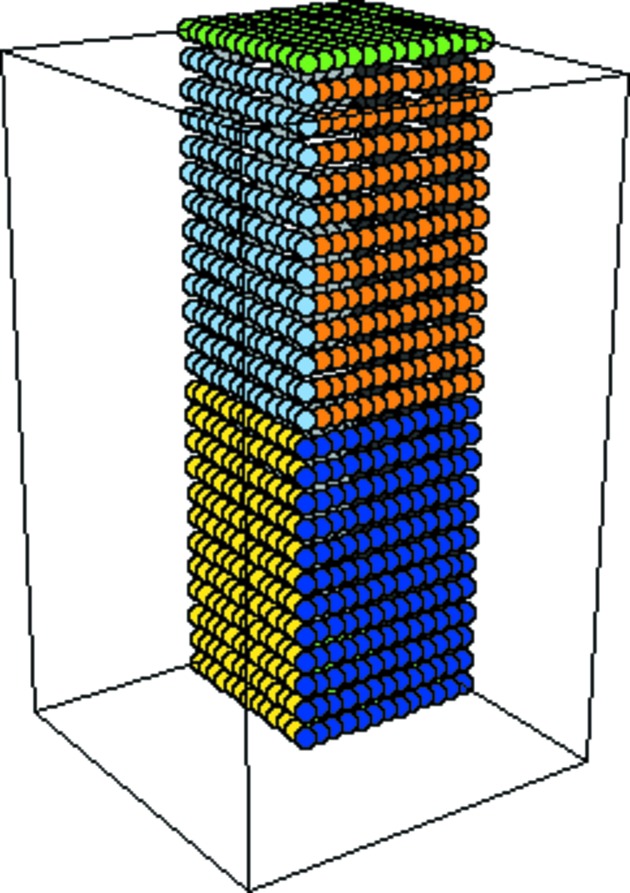
Visualization of redundant pairs of points at the surface of the DAU of space group 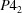 (No. 77). Colour-coded pairs of redundant points on the DAU surface are shown as they are detected in the sampling procedure described in §3.3. For example, the first point is coloured dark blue and the equivalent redundant point light blue. With the help of the colours it is immediately obvious how the redundant points are related and where to place the missing face cuts to obtain the exact DAU.

The exact DAU conditions shown in Table 2 ▸ were determined manually. Progress was greatly accelerated by visual tools developed specifically for this purpose. A full presentation of these tools is beyond the scope of this paper [Grosse-Kunstleve *et al.* (2003 ▸) includes pointers to the openly available implementation]. The main idea is to colour-code pairs of redundant points on the DAU surface as they are detected in the sampling procedure described in §3.3; for example, the first point is coloured dark blue and the equivalent redundant point light blue. A very simple colour-selection procedure using only a small palette of colours was found to be sufficient in practice. An example is shown in Fig. 2 ▸. We added the face- or edge-specific cuts one at a time, updating the visualization after each step. In this way we could determine exact DAU definitions in a matter of a few minutes for most space groups.

## 4. Results   

Table 2 ▸ defines exact DAUs for 230 *reference settings*, chosen to be compatible with the reference settings used in the IUCr symCIF dictionary (Brown, 2005 ▸). Using the change-of-basis transformation law of §3.1 in combination with the algorithms of Grosse-Kunstleve (1999 ▸), it is possible to automatically obtain an exact DAU for any setting.

Koch & Fischer (1974 ▸) and ITA §2.8 explain that the shape for a DAU is not uniquely determined and that the best choice is application specific. Similarly, the face- and edge-specific sub-conditions required for an exact DAU are also not uniquely determined. The choices we made for Table 2 ▸ aim at obtaining compact sets of sub-conditions, which is also expected to minimize the runtime needed for evaluating if a given point is inside the DAU. For the cubic space groups, we attempted to adopt the sub-conditions of Koch & Fischer (1974 ▸) but it turned out to be challenging in some cases. In six cases (space-group numbers 195, 198, 210, 220, 227, 228) the ITA shape conditions are incompatible with those of Koch & Fischer (1974 ▸). In some other cases their sub-conditions lead to complicated cut expressions. Using the approach of §3.4 it was only a small effort to determine simpler alternatives for Table 2 ▸.

For eight enantiomorphic space groups (the numbers are listed in the caption of Table 2 ▸) the exact DAU is defined through a change-of-basis transformation of the DAU of the enantiomorphic mate. The three remaining enantiomorphic pairs of space groups cannot be handled in this way because the ITA shape DAU conditions are pairwise incompatible. The change-of-basis matrices in Table 2 ▸ are expressed using the notation as defined in Zwart *et al.* (2008 ▸).

## 5. Conclusion   

Table 2 ▸ is the first complete and uniform definition of exact DAUs for all 230 space-group types. The table is concise owing to the geometric cut notation introduced in this work. At the same time, the cut expressions lend themselves to automatic processing, with results as demonstrated already in Grosse-Kunstleve *et al.* (2003 ▸). In the meantime we have found other practical uses in the context of the *PHENIX* suite (Adams *et al.*, 2010 ▸), such as the search for interactions between pairs of atoms (Grosse-Kunstleve *et al.*, 2004 ▸) and a bulk-solvent-mask determination procedure.

In this work we have used a manual approach for the determination of the face- and edge-specific sub-conditions required for exact DAUs. We believe an algorithmic approach is possible but will require significantly more initial effort than our manual approach. The cut plane formalism presented here could serve as a basis for future automation work.

## Figures and Tables

**Table 1:** Definition of geometric cut symbols used in Table 2 ▸ See Fig. 1 ▸ for geometric illustrations of the main symbols.

Symbol	*h*, *k*, *l*	*c*	Expression
*x*_1_	(1, 0, 0)	1	
*x*_0_			*x*_1_0
*x*_2_			*x*_1_/2
*x*_3_			*x*_1_/3
*x*_4_			*x*_1_/4
*x*_8_			*x*_1_/8
*x*_34_			*x*_1_3/4
*y*_1_	(0, 1, 0)	1	
*y*_0_			*y*_1_0
*y*_2_			*y*_1_/2
*y*_3_			*y*_1_/3
*y*_4_			*y*_1_/4
*y*_8_			*y*_1_/8
*z*_1_	(0, 0, 1)	1	
*z*_0_			*z*_1_0
*z*_2_			*z*_1_/2
*z*_3_			*z*_1_/3
*z*_4_			*z*_1_/4
*z*_6_			*z*_1_/6
*z*_8_			*z*_1_/8
*z*_12_			*z*_1_/12
*p*_1_	(1, 1, 0)	1	
*p*_0_			*p*_1_0
*p*_2_			*p*_1_/2
*p*_3_			*p*_1_/3
*p*_4_			*p*_1_/4
*m*_1_	(1, 1, 0)	1	
*m*_0_			*m*_1_0
*m*_2_			*m*_1_/2
*m*_4_			*m*_1_/4
*h*_1_	(1, 2, 0)	1	
*h*_0_			*h*_1_0
*k*_1_	(2, 1, 0)	1	
*k*_0_			*k*_1_0
*xz*_1_	(1, 0, 1)	1	
*xz*_0_			*xz*_1_0
*xz*_2_			*xz*_1_/2
*xz*_4_			*xz*_1_/4
*zx*_1_	(1, 0, 1)	1	
*zx*_0_			*zx*_1_0
*zx*_2_			*zx*_1_/2
*yz*_1_	(0, 1, 1)	1	
*yz*_0_			*yz*_1_0
*yz*_2_			*yz*_1_/2
*yz*_4_			*yz*_1_/4
*zy*_1_	(0, 1, 1)	1	
*zy*_0_			*zy*_1_0
*zy*_2_			*zy*_1_/2
*zy*_4_			*zy*_1_/4
*dy*_8_	(1, 1, 1)	1/8	
*tx*_0_	(2, 1, 1)	0	
*ty*_0_	(1, 2, 1)	0	
*tz*_2_	(2, 1, 1)	1/2	

*Primary* geometric cut symbols are defined directly by a normal and constant. *Secondary* geometric cut symbols are defined by a cut expression based on a primary symbol. Note that the normal vector of all cuts with a ‘0’ subscript in the geometric cut symbol is the opposite of the normal vector of the other cut planes in the same flock. This convention greatly reduces the number of minus signs in the exact DAU definitions of Table 2 ▸.

**Table 2:** Exact DAU definitions for the 230 space groups, based on the geometric cut definitions of Table 1 ▸ Following *International Tables*, Vol. B (Shmueli, 2001 ▸) Table A1.4.2.7, the first column, *n*:*c*, lists the space-group numbers and setting codes separated by a colon. For monoclinic space groups, the setting code ‘*b*’ indicates ‘unique axis *b*’; ‘*b*1’ indicates ‘unique axis *b*, cell choice 1’. For orthorhombic, tetragonal and cubic space groups, ‘2’ indicates ‘origin choice 2’. For rhombohedral space groups, ‘*h*’ indicates ‘hexagonal axes’. The conditions for space groups 78, 95, 145, 154, 170, 172, 181 and 213 are defined by change-of-basis operations transforming the conditions of the enantiomorphic mates.

*n*:*c*	Cuts
1	*x*_0_; +*x*_1_; *y*_0_; +*y*_1_; *z*_0_; +*z*_1_
2	*x*_0_(*y*_0_(*z*_2_) *y*_2_(*z*_2_)); *x*_2_(*y*_0_(*z*_2_) *y*_2_(*z*_2_)); *y*_0_; +*y*_1_; *z*_0_; +*z*_1_
3:*b*	*x*_0_; +*x*_1_; *y*_0_; +*y*_1_; *z*_0_(*x*_2_); *z*_2_(*x*_2_)
4:*b*	*x*_0_; +*x*_1_; *y*_0_; +*y*_1_; *z*_0_(*x*_0_(+*y*_2_) *x*_2_(+*y*_2_)); *z*_2_(*x*_0_(+*y*_2_) *x*_2_(+*y*_2_))
5:*b*1	*x*_0_(*z*_2_); *x*_2_(*z*_2_); *y*_0_; +*y*_2_; *z*_0_; +*z*_1_
6:*b*	*x*_0_; +*x*_1_; *y*_0_; *y*_2_; *z*_0_; +*z*_1_
7:*b*1	*x*_0_; +*x*_1_; *y*_0_(+*z*_2_); *y*_2_(+*z*_2_); *z*_0_; +*z*_1_
8:*b*1	*x*_0_; +*x*_1_; *y*_0_; *y*_4_(+*x*_2_); *z*_0_; +*z*_1_
9:*b*1	*x*_0_; +*x*_1_; *y*_0_(+*z*_2_); *y*_4_(+*z*_2_); *z*_0_; +*z*_1_
10:*b*	*x*_0_(*z*_2_); *x*_2_(*z*_2_); *y*_0_; *y*_2_; *z*_0_; +*z*_1_
11:*b*	*x*_0_; +*x*_1_; *y*_0_(*z*_0_(*x*_2_) *z*_2_(*x*_2_)); *y*_4_; *z*_0_; +*z*_1_
12:*b*1	*x*_0_(*z*_2_); *x*_2_(*z*_2_); *y*_0_; *y*_4_(*x*_4_(*z*_2_)); *z*_0_; +*z*_1_
13:*b*1	*x*_0_(*z*_0_(*y*_2_) *z*_4_); *x*_2_(*z*_0_(*y*_2_) *z*_4_); *y*_0_; +*y*_1_; *z*_0_; +*z*_2_
14:*b*1	*x*_0_(*y*_0_(*z*_2_)); +*x*_1_; *y*_0_(*x*_2_(*z*_2_)); *y*_4_(+*z*_2_); *z*_0_; +*z*_1_
15:*b*1	*x*_0_(*z*_4_); *x*_2_(*z*_4_); *y*_0_; +*y*_2_; *z*_0_(*y*_4_(*x*_4_)); *z*_2_(-*y*_4_(*x*_4_))
16	*x*_0_(*z*_2_); *x*_2_(*z*_2_); *y*_0_(*z*_2_); *y*_2_(*z*_2_); *z*_0_; +*z*_1_
17	*x*_0_(*z*_4_ *z*_1_3/4); *x*_2_(*z*_4_ *z*_1_3/4); *y*_0_(*z*_2_); *y*_2_(*z*_2_); *z*_0_; +*z*_1_
18	*x*_0_; *x*_2_(*y*_0_); *y*_0_; +*y*_2_; *z*_0_; +*z*_1_
19	*x*_0_; +*x*_2_; *y*_0_(*z*_2_); *y*_2_(*z*_2_); *z*_0_(+*y*_2_); +*z*_1_
20	*x*_0_(*z*_4_); *x*_2_(*z*_4_); *y*_0_; *y*_2_(*z*_0_); *z*_0_; +*z*_2_
21	*x*_0_(*z*_2_); *x*_4_(*y*_4_); *y*_0_(*z*_2_); *y*_2_(*z*_2_); *z*_0_; +*z*_1_
22	*x*_0_(*z*_2_); *x*_4_(*z*_4_ *z*_1_3/4); *y*_0_(*z*_2_); *y*_4_(*z*_4_ *z*_1_3/4); *z*_0_; +*z*_1_
23	*x*_0_; *x*_2_(*y*_0_); *y*_0_; *y*_2_(*z*_0_); *z*_0_; *z*_2_(*x*_0_)
24	*x*_0_(*y*_4_); *x*_2_(*y*_4_); *y*_0_(*z*_4_); *y*_2_(*z*_4_); *z*_0_(*x*_4_); *z*_2_(*x*_4_)
25	*x*_0_; *x*_2_; *y*_0_; *y*_2_; *z*_0_; +*z*_1_
26	*x*_0_; *x*_2_; *y*_0_(+*z*_2_); *y*_2_(+*z*_2_); *z*_0_; +*z*_1_
27	*x*_0_(+*z*_2_); *x*_2_(+*z*_2_); *y*_0_(+*z*_2_); *y*_2_(+*z*_2_); *z*_0_; +*z*_1_
28	*x*_0_(*y*_2_); *x*_4_; *y*_0_; +*y*_1_; *z*_0_; +*z*_1_
29	*x*_0_(+*z*_2_); *x*_4_(+*z*_2_); *y*_0_; +*y*_1_; *z*_0_; +*z*_1_
30	*x*_0_(*y*_2_); *x*_2_(*y*_2_); *y*_0_; +*y*_1_; *z*_0_; +*z*_2_
31	*x*_0_; *x*_2_; *y*_0_(+*z*_2_); *y*_2_(+*z*_2_); *z*_0_; +*z*_1_
32	*x*_0_; *x*_2_(*y*_0_); *y*_0_; +*y*_2_; *z*_0_; +*z*_1_
33	*x*_0_; +*x*_2_; *y*_0_(+*z*_2_); *y*_2_(+*z*_2_); *z*_0_; +*z*_1_
34	*x*_0_; *x*_2_(*y*_0_); *y*_0_; +*y*_2_; *z*_0_; +*z*_1_
35	*x*_0_; *x*_4_(*y*_4_); *y*_0_; *y*_2_; *z*_0_; +*z*_1_
36	*x*_0_; *x*_2_; *y*_0_; +*y*_2_; *z*_0_; +*z*_2_
37	*x*_0_(+*z*_2_); *x*_4_(*y*_4_); *y*_0_(+*z*_2_); *y*_2_(+*z*_2_); *z*_0_; +*z*_1_
38	*x*_0_; *x*_2_; *y*_0_; *y*_2_; *z*_0_; +*z*_2_
39	*x*_0_(+*z*_2_); *x*_2_(+*z*_2_); *y*_0_(+*z*_2_); *y*_4_; *z*_0_; +*z*_1_
40	*x*_0_(+*z*_2_); *x*_4_; *y*_0_(+*z*_2_); *y*_2_(+*z*_2_); *z*_0_; +*z*_1_
41	*x*_0_; *x*_2_(*y*_0_); *y*_0_; +*y*_2_; *z*_0_; +*z*_2_
42	*x*_0_; *x*_4_(+*z*_2_); *y*_0_; *y*_4_(+*z*_2_); *z*_0_; +*z*_1_
43	*x*_0_; *x*_4_(-*y*_0_(+*z*_2_)); *y*_0_; +*y*_4_; *z*_0_; +*z*_1_
44	*x*_0_; *x*_2_; *y*_0_; *y*_2_; *z*_0_; +*z*_2_
45	*x*_0_; *x*_2_(*y*_0_); *y*_0_; +*y*_2_; *z*_0_; +*z*_2_
46	*x*_0_(*y*_2_); *x*_4_; *y*_0_; +*y*_1_; *z*_0_; +*z*_2_
47	*x*_0_; *x*_2_; *y*_0_; *y*_2_; *z*_0_; *z*_2_
48:2	*x*_0_(*y*_0_(*z*_2_)); *x*_4_(*z*_4_ *z*_1_3/4); ~ *y*_4_(*z*_4_ *z*_1_3/4); *y*_4_(*z*_4_ *z*_1_3/4); *z*_0_; +*z*_1_
49	*x*_0_(*z*_4_); *x*_2_(*z*_4_); *y*_0_(*z*_4_); *y*_2_(*z*_4_); *z*_0_; *z*_2_
50:2	*x*_0_(*y*_2_); *x*_4_(*y*_4_ *y*_1_3/4); *y*_0_; +*y*_1_; *z*_0_(*y*_4_ *y*_1_3/4); *z*_2_(*y*_4_ *y*_1_3/4)
51	*x*_0_(*z*_2_); *x*_4_; *y*_0_; *y*_2_; *z*_0_; +*z*_1_
52	*x*_0_; +*x*_1_; *y*_0_(*x*_4_ *x*_34_); *y*_4_(*z*_4_); *z*_0_(*x*_2_); *z*_2_(*x*_2_)
53	*x*_0_; *x*_2_; *y*_0_; +*y*_1_; *z*_0_(*y*_2_); *z*_4_(*x*_4_)
54	*x*_0_(*z*_4_); *x*_2_(*z*_4_); *y*_0_(*x*_4_); *y*_2_(*x*_4_); *z*_0_; +*z*_2_
55	*x*_0_; *x*_2_(*y*_0_); *y*_0_; +*y*_2_; *z*_0_; *z*_2_
56	*x*_0_(*y*_2_(*z*_0_)); *x*_4_(*y*_4_ *y*_1_3/4); *y*_0_; +*y*_1_; *z*_0_; +*z*_2_
57	*x*_0_(*y*_2_); *x*_2_(*y*_2_); *y*_0_; +*y*_1_; *z*_0_(*y*_4_ *y*_1_3/4); *z*_4_
58	*x*_0_; *x*_2_(*y*_0_); *y*_0_; +*y*_2_; *z*_0_; *z*_2_
59:2	*x*_0_(*y*_0_(*z*_2_)); *x*_4_; ~ *y*_4_; *y*_4_; *z*_0_; +*z*_1_
60	*x*_0_(*z*_4_); *x*_2_(*z*_4_); *y*_0_; *y*_2_(*x*_0_(*z*_0_)); *z*_0_; +*z*_2_
61	*x*_0_; *x*_2_(*y*_0_(*z*_0_)); *y*_0_; +*y*_2_; *z*_0_; +*z*_2_
62	*x*_0_; *x*_2_(*y*_0_(*z*_0_)); *y*_0_(+*z*_2_); *y*_4_; *z*_0_; +*z*_1_
63	*x*_0_; *x*_2_; *y*_0_; +*y*_2_; *z*_0_(*y*_4_(*x*_4_)); *z*_4_
64	*x*_0_; *x*_4_(*z*_4_); *y*_0_; +*y*_2_; *z*_0_(*y*_4_); *z*_2_(+*y*_4_)
65	*x*_0_; *x*_4_(*y*_4_); *y*_0_; *y*_2_; *z*_0_; *z*_2_
66	*x*_0_(*z*_4_); *x*_4_(*y*_4_); *y*_0_(*z*_4_); *y*_2_(*z*_4_); *z*_0_; *z*_2_
67	*x*_0_; *x*_2_; *y*_0_(*x*_4_); *y*_4_; *z*_0_(*x*_4_); *z*_2_(*x*_4_)
68:2	*x*_0_(*z*_4_); *x*_2_(*z*_4_); *y*_0_(*x*_4_); *y*_4_(*z*_4_); *z*_0_(+*x*_2_ *y*_4_(*x*_4_)); +*z*_2_
69	*x*_0_; *x*_4_(*z*_4_); *y*_0_; *y*_4_(*z*_4_); *z*_0_; *z*_2_
70:2	*x*_0_(*y*_0_(*z*_2_)); *x*_8_(*z*_8_ *z*_1_5/8); ~ *y*_8_(*z*_1_3/8 *z*_1_7/8); *y*_8_(*z*_8_ *z*_1_5/8); *z*_0_; +*z*_1_
71	*x*_0_; *x*_4_(*y*_4_(*z*_4_)); *y*_0_; *y*_2_; *z*_0_; *z*_2_
72	*x*_0_(*z*_4_); *x*_4_(*y*_4_(*z*_4_)); *y*_0_(*z*_4_); *y*_2_(*z*_4_); *z*_0_; *z*_2_
73	*x*_0_(*y*_4_); *x*_4_(*z*_4_(*y*_4_)); *y*_0_(*z*_4_); *y*_2_(*z*_4_); *z*_0_; +*z*_2_
74	*x*_0_; *x*_4_(*z*_4_ *z*_1_3/4); *y*_0_(*z*_2_); *y*_4_; *z*_0_; +*z*_1_
75	*x*_0_(*y*_0_); *x*_2_; *y*_0_; *y*_2_(*x*_2_); *z*_0_; +*z*_1_
76	*x*_0_(+*z*_4_); *x*_2_(+*z*_4_); *y*_0_(+*z*_1_3/4); *y*_2_(+*z*_1_3/4); *z*_0_; +*z*_1_
77	*x*_0_(+*z*_2_); *x*_2_(+*z*_2_); *y*_0_(+*z*_2_); *y*_2_(+*z*_2_); *z*_0_; +*z*_1_
78	76 *a*, *b*, *c* + 1
79	*x*_0_(*y*_0_); *x*_2_; *y*_0_; *y*_2_(*x*_2_); *z*_0_; +*z*_2_
80	*x*_0_(*y*_2_); *x*_2_(*y*_2_); *y*_0_; +*y*_1_; *z*_0_; +*z*_4_
81	*x*_0_(*y*_0_(*z*_2_)); *x*_2_; *y*_0_; *y*_2_(*x*_2_(*z*_2_)); *z*_0_; +*z*_1_
82	*x*_0_(*z*_0_(*y*_0_)); *x*_2_(*y*_0_(*z*_4_)); *y*_0_; *y*_2_(*x*_0_(*z*_4_)); *z*_0_; *z*_2_(*y*_0_)
83	*x*_0_(*y*_0_); *x*_2_; *y*_0_; *y*_2_(*x*_2_); *z*_0_; *z*_2_
84	*x*_0_(*y*_0_(*z*_4_)); *x*_2_; *y*_0_; *y*_2_(*x*_2_(*z*_4_)); *z*_0_; *z*_2_
85:2	~ *x*_4_(~ *y*_4_); *x*_4_(*z*_0_(~ *y*_4_) *z*_2_(~ *y*_4_)); ~ *y*_4_; *y*_4_(*x*_4_); *z*_0_(*y*_0_(*x*_0_)); *z*_2_(*y*_0_(*x*_0_))
86:2	~ *x*_4_(~ *y*_4_(*z*_4_)); *x*_4_(*z*_0_(~ *y*_4_) *z*_2_(+~ *y*_4_)); ~ *y*_4_; *y*_4_(*x*_4_(*z*_4_)); *z*_0_(*y*_0_(*x*_0_)); *z*_2_(*y*_0_(*x*_0_))
87	*x*_0_(*y*_0_); *x*_2_; *y*_0_; *y*_2_(*x*_2_); *z*_0_; *z*_4_(*y*_4_(*x*_4_) *x*_2_(*y*_0_))
88:2	*x*_0_; *x*_4_; *y*_0_(*x*_0_(*z*_2_) | *x*_4_(+*z*_4_)); *y*_4_(*x*_0_(*z*_8_ *z*_1_5/8)); *z*_0_; +*z*_1_
89	*x*_0_(*p*_0_); *x*_2_; *y*_0_; *y*_2_(*x*_2_); *z*_0_(*p*_0_); *z*_2_(*p*_0_)
90	*x*_0_; *x*_2_(*y*_0_); *y*_0_; *y*_2_(*x*_0_); *z*_0_(*p*_0_); *z*_2_(*p*_0_)
91	*x*_0_(*z*_8_(*y*_0_)); +*x*_1_; *y*_0_; +*y*_1_; *z*_0_(*x*_2_); *z*_8_(*m*_1_)
92	*x*_0_; +*x*_1_; *y*_0_; +*y*_1_; *z*_0_(*p*_0_); *z*_8_(*y*_2_)
93	*x*_0_(*y*_2_); *x*_2_(*y*_2_); *y*_0_; +*y*_1_; *z*_0_(*y*_2_); *z*_4_(*p*_0_ *m*_1_)
94	*x*_0_(*y*_0_); *x*_2_(*z*_2_(*y*_2_)); *y*_0_(*z*_2_(*x*_0_)); +*y*_2_; *z*_0_(*p*_0_); *z*_2_(*p*_0_)
95	91 *a* + 1, *b*, *c*
96	*x*_0_; +*x*_1_; *y*_0_; +*y*_1_; *z*_0_(*p*_0_); *z*_8_(*x*_2_)
97	*x*_0_(*y*_0_); *x*_2_; *y*_0_; *y*_2_(*x*_2_); *z*_0_(*p*_0_); *z*_4_(*m*_2_)
98	*x*_0_(*y*_2_); *x*_2_(*y*_2_); *y*_0_; +*y*_1_; *z*_0_(*m*_1_ *p*_0_); *z*_8_(*y*_4_ *y*_1_3/4)
99	*x*_0_; *y*_2_; *z*_0_; +*z*_1_; *p*_0_
100	*x*_0_(*y*_0_); *y*_0_; *z*_0_; +*z*_1_; *m*_2_
101	*x*_0_(+*z*_2_); *y*_2_(+*z*_2_); *z*_0_; +*z*_1_; *p*_0_
102	*x*_0_(+*z*_2_); *y*_2_(+*z*_2_); *z*_0_; +*z*_1_; *p*_0_
103	*x*_0_(*y*_0_); *x*_2_; *y*_0_; *y*_2_(*x*_2_); *z*_0_; +*z*_2_
104	*x*_0_(*y*_0_); *x*_2_; *y*_0_; *y*_2_(*x*_2_); *z*_0_; +*z*_2_
105	*x*_0_; *x*_2_; *y*_0_; *y*_2_; *z*_0_; +*z*_2_
106	*x*_0_(*y*_0_); *x*_2_; *y*_0_; +*y*_2_; *z*_0_; +*z*_2_
107	*x*_0_; *y*_2_; *z*_0_; +*z*_2_; *p*_0_
108	*x*_0_(*y*_0_); *y*_0_; *z*_0_; +*z*_2_; *m*_2_
109	*x*_0_; *x*_2_; *y*_0_; *y*_2_; *z*_0_; +*z*_4_
110	*x*_0_(*y*_0_); *x*_2_; *y*_0_; +*y*_2_; *z*_0_; +*z*_4_
111	*x*_0_(*z*_2_); *y*_2_(*z*_2_); *z*_0_; +*z*_1_; *p*_0_
112	*x*_0_(*z*_4_ *z*_0_(*y*_0_)); *x*_2_(*z*_4_); *y*_0_(*z*_4_); *y*_2_(*z*_4_ *z*_0_(*x*_2_)); *z*_0_; +*z*_2_
113	*x*_0_(*y*_0_(*z*_2_)); *y*_0_; *z*_0_; +*z*_1_; *m*_2_
114	*x*_0_(*y*_0_); *x*_2_; *y*_0_; *y*_2_(*x*_2_(*z*_0_)); *z*_0_; +*z*_2_
115	*x*_0_; *x*_2_; *y*_0_; *y*_2_; *z*_0_(*p*_0_); *z*_2_(*p*_0_)
116	*x*_0_(*y*_2_); *x*_2_(*y*_2_ *z*_0_(*y*_2_)); *y*_0_; +*y*_1_; *z*_0_(*y*_2_ *y*_0_(*x*_0_)); *z*_4_(*m*_1_ *p*_0_)
117	*x*_0_(*z*_0_(*y*_0_) *z*_2_(*y*_0_)); *x*_2_(*y*_0_); *y*_0_; +*y*_2_; *z*_0_(*m*_2_); *z*_2_(*m*_2_)
118	*x*_0_(*y*_2_); *x*_2_(*y*_2_); *y*_0_; +*y*_1_; *z*_0_(*y*_2_(*x*_2_) *x*_0_(*y*_0_)); *z*_4_(~ *p*_2_ *m*_2_)
119	*x*_0_; *x*_2_; *y*_0_; *y*_2_; *z*_0_(*p*_0_); *z*_4_(*m*_2_)
120	*x*_0_(*z*_0_(*y*_0_)); *x*_2_(*y*_0_); *y*_0_; +*y*_2_; *z*_0_(*m*_2_); *z*_4_(*p*_0_)
121	*x*_0_; *y*_2_(*x*_0_(*z*_4_)); *z*_0_; *z*_2_(*x*_0_); *p*_0_
122	*x*_0_(*y*_2_ *z*_0_(*y*_0_)); *x*_2_(*y*_2_); *y*_0_; +*y*_1_; *z*_0_(*y*_2_(*x*_2_)); *z*_8_(*y*_4_ *y*_1_3/4)
123	*x*_0_; *y*_2_; *z*_0_; *z*_2_; *p*_0_
124	*x*_0_(*y*_0_); *x*_2_; *y*_0_; *y*_2_(*x*_2_); *z*_0_; *z*_4_(*p*_0_)
125:2	~ *x*_4_(~ *y*_4_); ~ *y*_4_; *z*_0_(*p*_0_); *z*_2_(*p*_0_); *m*_0_
126:2	~ *x*_4_( ~ *y*_4_); *x*_4_; ~ *y*_4_; *y*_4_(*x*_4_); *z*_0_(*y*_0_(*x*_0_) *x*_4_(~ *y*_4_)); *z*_4_(*p*_0_)
127	*x*_0_(*y*_0_); *y*_0_; *z*_0_; *z*_2_; *m*_2_
128	*x*_0_(*y*_0_); *x*_2_; *y*_0_; *y*_2_(*x*_2_); *z*_0_; *z*_4_(*m*_2_)
129:2	~ *x*_4_; *y*_4_; *z*_0_(*m*_0_); *z*_2_(*m*_0_); *p*_0_
130:2	~ *x*_4_(~ *y*_4_); *x*_4_(*z*_0_(~ *y*_4_)); ~ *y*_4_; *y*_4_(*x*_4_); *z*_0_(*y*_0_(*x*_0_)); *z*_4_(*m*_0_)
131	*x*_0_; *x*_2_; *y*_0_; *y*_2_; *z*_0_; *z*_4_(*p*_0_)
132	*x*_0_(*z*_4_); *y*_2_(*z*_4_); *z*_0_; *z*_2_; *p*_0_
133:2	~ *x*_4_; *x*_4_(*z*_0_ | ~ *y*_4_); ~ *y*_4_; +*y*_4_; *z*_0_(*y*_0_(*x*_0_)); *z*_4_(*p*_0_)
134:2	~ *x*_4_(*z*_4_); ~ *y*_4_(*z*_4_); *z*_0_(*p*_0_); *z*_2_(*p*_0_); *m*_0_
135	*x*_0_(*z*_4_(*y*_0_)); *x*_2_(*y*_0_); *y*_0_; +*y*_2_; *z*_0_; *z*_4_(*m*_2_)
136	*x*_0_(*z*_4_); *y*_2_(*z*_4_(*x*_0_)); *z*_0_; *z*_2_; *p*_0_
137:2	~ *x*_4_; *x*_4_; ~ *y*_4_; *y*_4_; *z*_0_(*y*_0_(*x*_0_)); *z*_4_(*m*_0_)
138:2	~ *x*_4_; *y*_4_(~ *x*_4_(*z*_4_)); *z*_0_(*m*_0_); *z*_2_(*m*_0_ ~ *x*_4_(*y*_4_)); *p*_0_
139	*x*_0_; *y*_2_; *z*_0_; *z*_4_(*m*_2_); *p*_0_
140	*x*_0_(*y*_0_); *y*_0_; *z*_0_; *z*_4_(*p*_0_); *m*_2_
141:2	*x*_0_; *x*_2_; ~ *y*_4_; *y*_4_; *z*_0_(*y*_0_); *z*_8_(*p*_4_)
142:2	*x*_0_(*z*_8_( ~ *y*_4_) *z*_0_(*y*_0_)); *x*_2_( ~ *y*_4_); ~ *y*_4_; +*y*_4_; *z*_0_(*x*_4_); *z*_8_(*m*_4_)
143	*x*_0_(*y*_0_); *y*_0_; *z*_0_; +*z*_1_; *k*_1_; *m*_1_(*h*_1_ | *k*_1_); *h*_1_
144	*x*_0_; +*x*_1_; *y*_0_; +*y*_1_; *z*_0_; +*z*_3_
145	144 *b*, *a*, *c*
146:*h*	*x*_0_(*y*_0_); *y*_0_; *z*_0_; +*z*_3_; *k*_1_; *m*_1_(*h*_1_ | *k*_1_); *h*_1_
147	*x*_0_(*y*_0_); *y*_0_; *z*_0_(*p*_0_(*y*_0_)); *z*_2_(*p*_0_(*y*_0_)); *k*_1_; *m*_1_(*h*_1_ | *k*_1_); *h*_1_
148:*h*	*x*_0_(*y*_0_); *y*_0_; *z*_0_(*p*_0_(*y*_0_)); *z*_6_(*h*_0_(*x*_3_) | *k*_0_(*y*_0_ | *m*_1_)); *k*_1_; *m*_1_(*h*_1_ | *k*_1_); *h*_1_
149	*x*_0_(*y*_0_); *y*_0_; *z*_0_(*h*_0_ | *k*_0_); *z*_2_(*h*_0_ | *k*_0_); *k*_1_; *m*_1_(*h*_1_ | *k*_1_); *h*_1_
150	*x*_0_(*y*_0_); *y*_0_; *z*_0_(*p*_0_); *z*_2_(*p*_0_); *k*_1_; *m*_1_(*h*_1_ | *k*_1_); *h*_1_
151	*x*_0_; +*x*_1_; *y*_0_; +*y*_1_; *z*_0_(*h*_0_ | *h*_1_); *z*_6_(*k*_0_ | *k*_1_)
152	*x*_0_; +*x*_1_; *y*_0_; +*y*_1_; *z*_0_(*p*_0_); *z*_6_(*p*_0_)
153	*x*_0_; +*x*_1_; *y*_0_; +*y*_1_; *z*_0_(*h*_0_ | *h*_1_); *z*_6_(*x*_0_(*y*_0_) *m*_1_)
154	152 *b*, *a*, *c*
155:*h*	*x*_0_(*y*_0_); *y*_0_; *z*_0_(*p*_0_); *z*_6_(*x*_3_ ~ *p*_3_); *k*_1_; *m*_1_(*h*_1_ | *k*_1_); *h*_1_
156	*z*_0_; +*z*_1_; *h*_0_; *m*_1_; *k*_0_
157	*y*_0_; *z*_0_; +*z*_1_; *k*_1_; *m*_1_(*y*_3_); *p*_0_
158	*x*_0_(*y*_0_); *y*_0_; *z*_0_; +*z*_2_; *k*_1_; *m*_1_(*h*_1_ | *k*_1_); *h*_1_
159	*x*_0_(*y*_0_); *y*_0_; *z*_0_; +*z*_2_; *k*_1_; *m*_1_(*h*_1_ | *k*_1_); *h*_1_
160:*h*	*z*_0_; +*z*_3_; *h*_0_; *m*_1_; *k*_0_
161:*h*	*x*_0_(*y*_0_); *y*_0_; *z*_0_; +*z*_6_; *k*_1_; *m*_1_(*h*_1_ | *k*_1_); *h*_1_
162	*y*_0_; *z*_0_(*h*_0_); *z*_2_(*h*_0_); *k*_1_; *m*_1_(*y*_3_); *p*_0_
163	*x*_0_(*y*_0_); *y*_0_; *z*_0_(*p*_0_(*y*_0_)); *z*_4_(*h*_0_ | *k*_0_); *k*_1_; *m*_1_(*h*_1_ | *k*_1_); *h*_1_
164	*y*_0_(*z*_2_); *z*_0_; +*z*_1_; *k*_1_; *h*_0_
165	*x*_0_(*y*_0_); *y*_0_; *z*_0_(*p*_0_(*y*_0_)); *z*_4_(*p*_0_); *k*_1_; *m*_1_(*h*_1_ | *k*_1_); *h*_1_
166:*h*	*z*_0_(*p*_0_); *z*_6_(*x*_3_); *h*_0_; *m*_1_; *k*_0_
167:*h*	*x*_0_(*y*_0_); *y*_0_; *z*_0_(*p*_0_(*y*_0_)); *z*_12_(*y*_3_ *p*_3_); *k*_1_; *m*_1_(*h*_1_ | *k*_1_); *h*_1_
168	*y*_0_; *z*_0_; +*z*_1_; *k*_1_; *m*_1_(*y*_3_); *p*_0_(*y*_0_)
169	*x*_0_; +*x*_1_; *y*_0_; +*y*_1_; *z*_0_; +*z*_6_
170	169 *b*, *a*, *c*
171	*x*_1_(*y*_2_); *y*_0_(*x*_2_); *z*_0_; +*z*_3_; *p*_0_(*y*_2_)
172	171 *b* + 1, *a* + 1, *c*
173	*x*_0_(*y*_0_); *y*_0_; *z*_0_; +*z*_2_; *k*_1_; *m*_1_(*h*_1_ | *k*_1_); *h*_1_
174	*x*_0_(*y*_0_); *y*_0_; *z*_0_; *z*_2_; *k*_1_; *m*_1_(*h*_1_ | *k*_1_); *h*_1_
175	*y*_0_; *z*_0_; *z*_2_; *k*_1_; *m*_1_(*y*_3_); *p*_0_(*y*_0_)
176	*x*_0_(*y*_0_); *y*_0_; *z*_0_(*p*_0_(*y*_0_)); *z*_4_; *k*_1_; *m*_1_(*h*_1_ | *k*_1_); *h*_1_
177	*y*_0_; *z*_0_(*h*_0_); *z*_2_(*h*_0_); *k*_1_; *m*_1_(*y*_3_); *p*_0_(*y*_0_)
178	*x*_0_; +*x*_1_; *y*_0_; +*y*_1_; *z*_0_(*p*_0_); *z*_12_(*h*_0_ | *h*_1_)
179	*x*_0_; +*x*_1_; *y*_0_; +*y*_1_; *z*_0_(*p*_0_); *z*_12_(*m*_1_ *x*_0_(*y*_0_))
180	*x*_1_(*y*_2_); *y*_0_(*x*_2_); *z*_0_(*k*_1_); *z*_6_(*h*_0_); *p*_0_(*y*_2_)
181	180 *b* + 1, *a* *b*, *c* + 1/6
182	*x*_0_(*y*_0_); *y*_0_; *z*_0_(*p*_0_); *z*_4_(*h*_0_ | *k*_0_); *k*_1_; *m*_1_(*h*_1_ | *k*_1_); *h*_1_
183	*y*_0_; *z*_0_; +*z*_1_; *k*_1_; *h*_0_
184	*y*_0_; *z*_0_; +*z*_2_; *k*_1_; *m*_1_(*y*_3_); *p*_0_(*y*_0_)
185	*y*_0_; *z*_0_; +*z*_2_; *k*_1_; *m*_1_(*y*_3_); *p*_0_
186	*y*_0_(+*z*_2_); *z*_0_; +*z*_1_; *k*_1_; *h*_0_
187	*z*_0_; *z*_2_; *h*_0_; *m*_1_; *k*_0_
188	*x*_0_(*y*_0_); *y*_0_; *z*_0_(*h*_0_ | *k*_0_); *z*_4_; *k*_1_; *m*_1_(*h*_1_ | *k*_1_); *h*_1_
189	*y*_0_; *z*_0_; *z*_2_; *k*_1_; *m*_1_(*y*_3_); *p*_0_
190	*x*_0_(*y*_0_); *y*_0_; *z*_0_(*p*_0_); *z*_4_; *k*_1_; *m*_1_(*h*_1_ | *k*_1_); *h*_1_
191	*y*_0_; *z*_0_; *z*_2_; *k*_1_; *h*_0_
192	*y*_0_; *z*_0_; *z*_4_(*h*_0_); *k*_1_; *m*_1_(*y*_3_); *p*_0_(*y*_0_)
193	*y*_0_; *z*_0_(*h*_0_); *z*_4_; *k*_1_; *m*_1_(*y*_3_); *p*_0_
194	*z*_0_(*p*_0_); *z*_4_; *h*_0_; *m*_1_; *k*_0_
195	*z*_0_(*y*_2_ *x*_2_); *m*_1_(*y*_2_); *zy*_0_(*zx*_0_); *zx*_0_
196	*p*_0_(*m*_2_); ~ *xz*_2_(*zy*_0_); *zx*_2_(*yz*_0_); *yz*_0_; *zy*_0_
197	*z*_0_(*x*_2_); *p*_0_(*zy*_0_); +*m*_1_; *zy*_0_
198	*x*_0_(*y*_0_); *x*_2_; *y*_2_(+*z*_0_ *x*_2_(+*z*_2_)); *zx*_2_(*m*_2_); *zx*_0_(*p*_0_); *yz*_0_; *zy*_0_
199	*x*_2_(*y*_4_); *y*_2_(*z*_4_); *z*_0_(*x*_4_); *zx*_0_(*zy*_0_(+*x*_2_)); *zy*_0_
200	*x*_2_; *y*_2_; *z*_0_; *zx*_0_(*zy*_0_); *zy*_0_
201:2	~ *z*_4_(*x*_4_); *p*_0_(*zy*_0_(*x*_0_)); *m*_2_(*zy*_0_(*x*_2_)); *zy*_0_
202	*z*_0_; *p*_0_(*x*_4_); ~ *xz*_2_(*zy*_0_); *zy*_0_
203:2	*p*_0_(*zy*_0_(*x*_0_)); *m*_4_(*zy*_0_ | *yz*_4_( ~ *z*_4_)); *zy*_0_; *yz*_4_
204	*x*_2_; *z*_0_; *p*_0_(*zy*_0_(*x*_4_)); *zy*_0_
205	*x*_2_(*z*_0_(*zy*_0_)); *y*_2_(*zy*_0_); *z*_0_; *zx*_0_(*zy*_0_); *zy*_0_
206	*z*_0_(*x*_4_); *zx*_0_(*zy*_0_); ~ *xz*_2_; *zy*_0_; ~ *yz*_2_(*zx*_0_)
207	*z*_0_(*x*_2_); *p*_0_; *m*_1_(*p*_0_); *zy*_0_(*x*_2_)
208	*zx*_0_(*zy*_0_); *xz*_0_(*yz*_0_); *zx*_2_(*y*_4_); ~ *xz*_2_(*y*_4_); *zy*_0_; *yz*_0_; *zy*_2_(*x*_4_); ~ *yz*_2_(*x*_4_)
209	*p*_0_(*z*_0_); *m*_2_(*z*_0_); *zy*_0_; *yz*_0_(*zy*_0_)
210	*y*_8_( ~ *xz*_4_); *z*_8_(*m*_4_); *p*_0_(*zx*_0_); *m*_2_(~ *xz*_2_); *yz*_0_(*z*_0_); *zx*_0_; ~ *xz*_2_
211	*z*_0_(*p*_0_); *zx*_0_(*zy*_0_); ~ *xz*_2_(*y*_4_); *zy*_0_; ~ *yz*_2_(*x*_4_)
212	*zx*_2_; *yz*_0_(*zx*_2_); ~ *yz*_2_(*tx*_0_); *tx*_0_(*x*_8_); *ty*_0_(*y*_8_); *tz*_2_(*x*_1_3/8)
213	212 *b*, *c* + 1/2, *a* 1/2
214	*x*_8_(~ *yz*_4_); *y*_8_(~ *xz*_4_); ~ *y*_8_(~ *zx*_1_/4); *zx*_0_(*zy*_0_); *zy*_0_; ~ *zy*_4_(*y*_0_); *dy*_8_(~ *p*_4_)
215	*z*_0_(*x*_2_); *p*_0_; *m*_1_; *zy*_0_
216	*p*_0_; *m*_2_; *zy*_0_; *yz*_0_
217	*x*_2_(*z*_0_(*y*_4_)); *z*_0_; *p*_0_; *zy*_0_
218	*x*_2_(*z*_0_(*y*_4_)); *y*_2_(*z*_0_(*x*_4_)); *z*_0_; *zx*_0_(*zy*_0_); *zy*_0_
219	*p*_0_; *m*_2_(*p*_0_(*z*_0_)); *zy*_0_; *yz*_0_(*p*_0_ | *zy*_0_(*x*_4_))
220	*x*_4_(*z*_0_(*y*_1_3/8)); *x*_2_; *y*_4_(*x*_2_(*z*_8_)); *y*_2_(*z*_4_); *z*_0_; *zx*_0_(*zy*_0_); *zy*_0_
221	*x*_2_; *z*_0_; *p*_0_; *zy*_0_
222:2	*x*_34_(*z*_4_(*y*_2_) | *zy*_0_); *z*_4_; *p*_0_(*zy*_0_(*z*_2_)); *zy*_0_
223	*z*_0_; *zx*_0_(*zy*_0_); ~ *xz*_2_(*y*_4_); *zy*_0_; ~ *yz*_2_(*x*_4_)
224:2	*p*_0_; ~ *xz*_1_(*y*_2_); *zx*_2_(*y*_2_); ~ *yz*_2_; *zy*_0_
225	*z*_0_; *p*_0_; *m*_2_; *zy*_0_
226	*z*_0_; *p*_0_; *m*_2_(*p*_0_); *zy*_0_(*x*_4_)
227:2	*y*_0_(*xz*_0_); *p*_0_; *m*_4_; *yz*_4_; *zy*_0_
228:2	*y*_0_(*zx*_1_/4); *p*_0_(*zy*_0_); *m*_4_; *yz*_4_(*zy*_0_(*x*_8_)); *zy*_0_
229	*z*_0_; *p*_0_; ~ *xz*_2_(*y*_4_); *zy*_0_
230	*x*_8_(~ *zy*_4_ ~ *yz*_4_); ~ *x*_8_(*y*_0_(*z*_4_)); *y*_8_(~ *xz*_4_); ~ *y*_8_(~ *zx*_1_/4); *z*_4_(*y*_0_); *zx*_0_; *xz*_0_(*z*_0_); *zy*_0_(*zx*_0_); *yz*_0_

## Appendix A Example step-by-step interpretation of a geometric cut expression

Equation (9) in §2, which appears for space group  (No. 112) in Table 2 ▸, was shown as an example of a geometric cut expression:

The shape cut in this example is . According to Table 1 ▸ and employing equation (7), this geometric cut symbol expands to . Use of equation (1) yields , which simplifies to . The outer pair of parentheses encloses a face cut expression that applies only if  [see text following equation (8) in §2]. The first part of the face cut expression, , translates to  [lookup in Table 1 ▸, use of equations (8) and (1), and simplification]. The second part of the face cut expression, , translates to . The logical conjunction symbol ‘&’ (defined in §2) indicates that a point in the plane is in the DAU only if both  *and* , with *and* in the Boolean sense. The inner pair of parentheses encloses an edge cut expression specific to the (0, *y*, 0) line defined by the conditions  and . Lookup in Table 1 ▸, use of equations (7), (5) and (1), and simplification lead to the sub-condition . In combination with the  shape cut of space group , this means that only the point (0, 0, 0) on the (0, *y*, 0) line is in the DAU.

Graphical illustrations of the DAU conditions are available at http://cci.lbl.gov/asu_gallery/. An expanded DAU notation that is more similar to the notation of ITA is shown along with the graphical illustrations.
